# Supplementary material for: α-Fe2O3/, Co3O4/, and CoFe2O4/MWCNTs/Ionic Liquid Nanocomposites as High-Performance Electrocatalysts for the Electrocatalytic Hydrogen Evolution Reaction in a Neutral Medium
Source: Int J Mol Sci. 2024 Jun 27;25(13):7043. doi: 10.3390/ijms25137043 (PMC11240971; doi:10.3390/ijms25137043)
Supplement: Supplementary file 1 [file ijms-25-07043-s001.zip › ijms-3038642-supplementary.pdf]

International Journal of *Molecular Sciences*

MDPI

Article

α-Fe_2O_3/, Co_3O_4/, and $CoFe_2O_4$/MWCNTs/Ionic Liquid Nanocomposites as High-Performance Electrocatalysts for the Electrocatalytic Hydrogen Evolution Reaction in a Neutral Medium

José Ibarra [1], María Jesus Aguirre [2,3], Rodrigo del Río [1,2], Rodrigo Henriquez [4], Ricardo Faccio [5], Enrique A. Dalchiele [6], Roxana Arce [2,7,*] and Galo Ramírez [1,2,*]

1 Departamento de Química Inorgánica, Facultad de Química, Pontificia Universidad Católica de Chile, Av. Vicuña Mackenna 4860, Casilla 306, Correo 22, Santiago 8331150, Chile; jfibarra@uc.cl (J.I.); rdelrioq@uc.cl (R.d.R.)
2 Millennium Institute on Green Ammonia as Energy Vector (MIGA), Av. Vicuña Mackenna 4860, Macul, Santiago 7820436, Chile; maria.aguirre@usach.cl
3 Departamento Química de los Materiales, Facultad de Química y Biologia, Universidad de Santiago de Chile, Av. B O'Higgins 3363, Estación Central, Santiago 9170022, Chile
4 Instituto de Química, Facultad de Ciencias, Pontificia Universidad Católica de Valparaíso, Av. Brasil 2950, Valparaíso 2362807, Chile; rodrigo.henriquez@pucv.cl
5 Área Física & Centro NanoMat, DETEMA, Facultad de Química, Universidad de la República, Av. Gral. Flores 2124, CC 1157, Montevideo 11800, Uruguay; rfaccio@fq.edu.uy
6 Instituto de Física, Facultad de Ingeniería, Universidad de la República, Herrera y Reissig 565, C.C. 30, Montevideo 11000, Uruguay; dalchiel@hotmail.it
7 Departamento de Ciencias Químicas, Facultad de Ciencias Exactas, Universidad Andrés Bello, Av. República 275, Santiago 8370146, Chile
* Correspondence: roxana.arce@unab.cl (R.A.); gramirezj@uc.cl (G.R.)

Abstract: Transition metal oxides are a great alternative to less

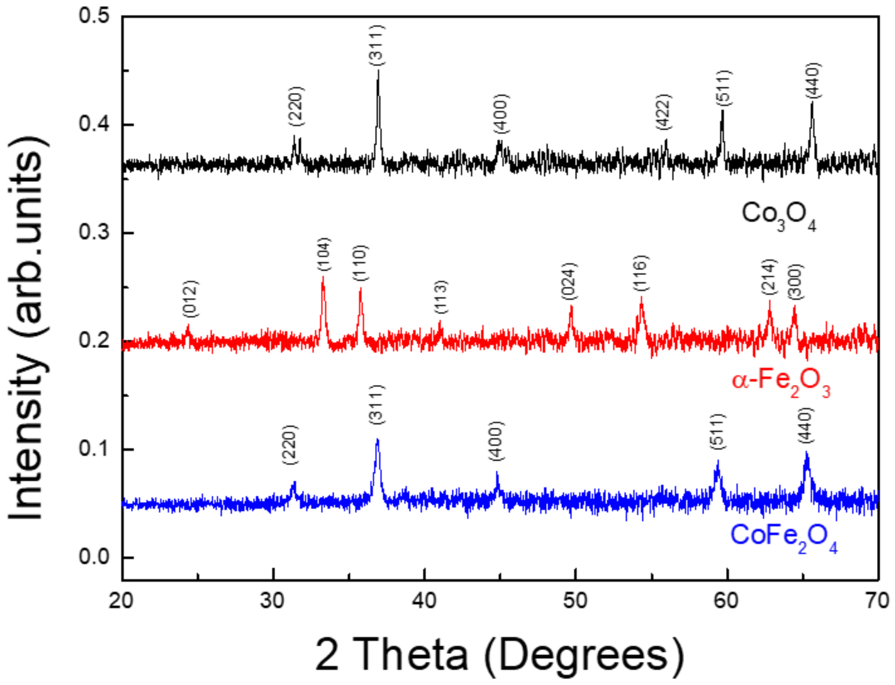

Figure S1. XRD diffractogram for the synthesized transition metal oxides

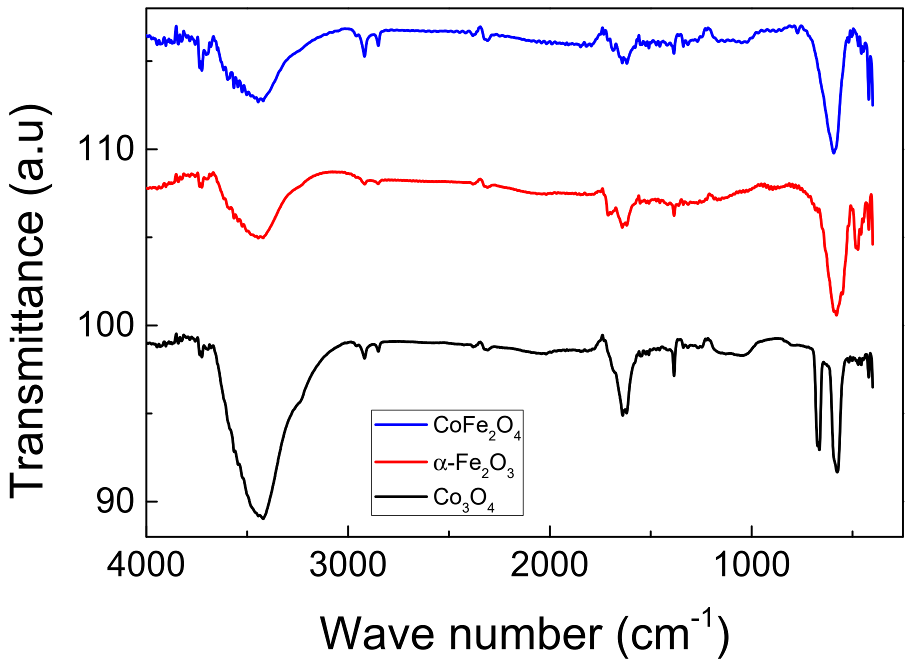

Figure S2. FT-IR spectra for the transition metal oxides synthesized

The infrared spectra of the investigated metal oxides (Figure X) exhibit distinct features reflective of their unique crystal structures and compositions. Hematite (α-Fe_2O_3) displays characteristic bands at 450-550 cm^{-1} and 550-650 cm^{-1}, attributed to Fe-O stretching and bending vibrations, respectively. The broader and more complex nature of the higher frequency band is likely due to contributions from multiple vibrational modes, including lattice vibrations and overtones. In contrast, both cobalt ferrite ($CoFe_2O_4$) and cobalt oxide (Co_3O_4), with their spinel structures, exhibit a prominent band at 550-600 cm^{-1} corresponding to M-O stretching vibrations in tetrahedral sites. However, a key distinguishing factor is the octahedral M-O stretching band, observed at 350-450 cm^{-1} for $CoFe_2O_4$ and at a higher frequency of 650-700 cm^{-1} for Co_3O_4, due to the exclusive presence of Co^{3+} ions in those sites.

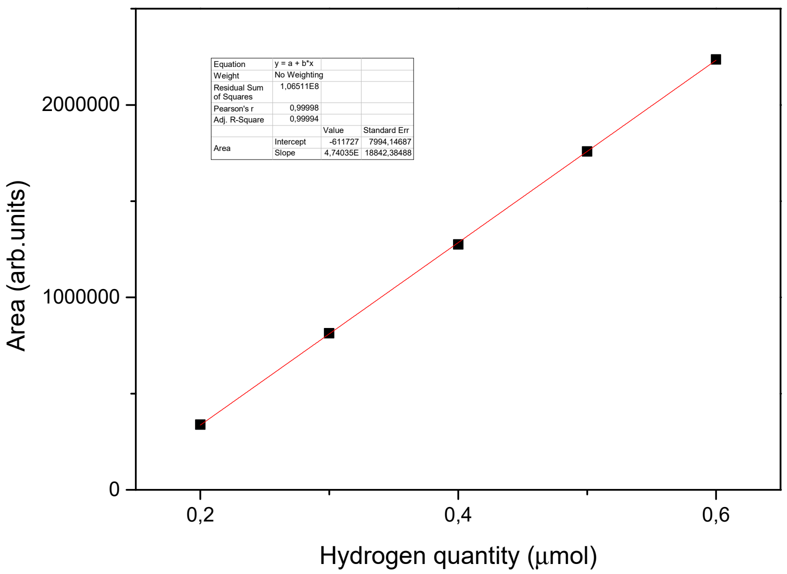

Figure S3. Chromatographic calibration curve to quantify Hydrogen
